# Comparison of temporal muscle fascia and tragal cartilage perichondrium in endoscopic type 1 tympanoplasty with limited elevation of tympanomeatal flap^☆^

**DOI:** 10.1016/j.bjorl.2019.06.014

**Published:** 2019-07-27

**Authors:** Kadir Özdamar, Alper Sen

**Affiliations:** a Private Lotus Hospital, Department of Otorhinolaryngology, Head and Neck Surgery, Şanlıurfa, Turkey; b Harran University, Medical Faculty, Department of Otorhinolaryngology, Head and Neck Surgery, Şanlıurfa, Turkey

**Keywords:** Endoscopic tympanoplasty, Tympanomeatal flap, Perichondrium, Temporal muscle fascia, Timpanoplastia endoscópica, Retalho timpanomeatal, Pericôndrio, Fáscia do músculo temporal

## Abstract

**Introduction:**

Elevation of tympanomeatal flap is one of the basic steps of tympanoplasty. A satisfactory level of anatomic and functional success can be achieved by using different grafts with limited tympanomeatal flap elevation.

**Objectives:**

We aimed to compare the anatomic and functional success of tragal cartilage perichondrium and temporal muscle fascia in cases of endoscopic type 1 tympanoplasty performed with limited tympanomeatal flap elevation.

**Methods:**

In total, 81 cases (33 females, 48 males, mean age 22.1 ± 10.1 years, interval 18–49 years) which underwent transcanal endoscopic type 1 tympanoplasty with limited elevation of tympanomeatal flap were included the present study. All cases were divided into two groups as tragal cartilage perichondrium (group A) and temporal muscle fascia (group B). The comparison of the groups were made considering the pre- and postoperative air-bone gap and the tympanic membrane status.

**Results:**

There was no statistically significant difference between Group A and Group B in preoperative and in postoperative air-bone gap values (*p* = 0.608 and 0.529, respectively). In Group A and B, postoperative air-bone gap values demonstrated significant decrease compared to the preoperative values (*p* = 0.0001). Group A and group B did not demonstrate significant differences between postoperative improvements of air-bone gap values (*p* = 0.687). Graft retention success was 92.6% in group A while it was 90.0% in group B. There was no statistically significant difference between the groups in terms of graft retention success (*p* = 0.166).

**Conclusion:**

In accordance with the results of this study, we believe that both tragal cartilage perichondrium and temporal muscle fascia, and also in limited tympanomeatal flap elevation in endoscopic tympanoplasty are all eligible for result in safe and successful surgery.

## Resumo

**Introdução:**

O descolamento do retalho timpanomeatal é uma das etapas básicas da timpanoplastia. Um nível satisfatório de sucesso na restauração anatômica e funcional pode ser alcançado com o uso de diferentes enxertos e descolamento limitado do retalho timpanomeatal.

**Objetivos:**

Comparar os resultados anatômicos e funcionais entre o uso de pericôndrio de cartilagem tragal e de fáscia do músculo temporal em timpanoplastias endoscópicas tipo 1 feitas com descolamento limitado do retalho timpanomeatal.

**Método:**

Foram incluídos no estudo 81 pacientes (33 mulheres, 48 ​​homens, média de 22,1 ± 10,1 anos, variação de 18-49 anos), submetidos a timpanoplastia endoscópica transcanal tipo 1 com descolamento limitado do retalho timpanomeatal. Todos os casos foram divididos em dois grupos: pericôndrio da cartilagem tragal (grupo A) e fáscia do músculo temporal (grupo B). Na comparação dos grupos consideraram-se o *gap* aéreo-ósseo, pré e pós-operatório e a condição da membrana timpânica.

**Resultados:**

Não houve diferença estatisticamente significante entre os grupos A e B no pré e pós-operatório (*p* =  0,608 e 0,529, respectivamente). Nos grupos A e B, os valores do gap aéreo-ósseo no pós-operatório demonstraram redução significante em relação aos valores pré-operatórios (*p* =  0,0001). Os grupos A e B não demonstraram diferenças significantes entre as medidas pré e pós-operatórias dos valores dos gaps (*p* =  0,687). O sucesso da retenção do enxerto foi de 92,6% no grupo A, enquanto no grupo B foi de 90,0%, não ocorreu diferença estatisticamente significante entre os grupos (*p* =  0,166).

**Conclusão:**

De acordo com os resultados deste estudo, acreditamos que tanto o pericôndrio da cartilagem tragal como a fáscia do músculo temporal, usados com descolamento limitado do retalho timpanomeatal na timpanoplastia endoscópica, são elegíveis para uma cirurgia segura e bem-sucedida.

## Introduction

Tympanoplasty is a surgical procedure performed to correct loss of hearing by repairing tympanic membrane perforation.^1^, ^2^ Tympanoplasty can be implemented using different techniques and grafts. Although there is no consensus on surgical technique and graft selection, the experience and preferences of the surgeon are paramount in the repair selection.^3^

Developments related to tympanoplasty continue rapidly in modern otologic surgery. In the literature there is a considerably high number of studies related to tympanoplasty. Tympanoplasty techniques and key points in choosing graft materials constitute the main topics of these studies.^4^, ^5^, ^6^

With the development and widespread use of modern tympanoplasty techniques, the success of tympanoplasty rises over 90%.^7^, ^8^ Modern tympanoplasty techniques started with the usage of microscopes, and today the endoscopic tympanoplasty method is defined in accordance with the principles of minimally invasive surgery. The usage of endoscopic tympanoplasty method started in the 1990s and has become popular in otology. Endoscopically, the tympanomeatal flap can be elevated through the transcanal route, whereas tympanoplasty can also be performed without the tympanomeatal flap being elevated. The anatomic structures in the middle ear, the anterior and posterior epitympanic space, sinus tympani and the fascial recess can be viewed with better imaging by the endoscopic method.^9^

Elevation of tympanomeatal flap is one of the basic steps of tympanoplasty.^10^ Different incisions have been reported in the literature for the elevation of a tympanomeatal flap. Elevation of a superiorly based flap with radial incision is frequently the most preferred option.^11^, ^12^ The amount of tympanomeatal flap to be elevated varies depending on the size of the perforation. Ayache^11^ defined different types of tympanomeatal flap incision according to perforation size and location. A satisfactory level of anatomic and functional success can be achieved by using different grafts with limited tympanomeatal flap elevation.

In this study, the anatomic and functional successes of tragal cartilage perichondrium and temporal muscle fascia were compared in cases of endoscopic type 1 tympanoplasty performed with limited tympanomeatal flap elevation.

## Methods

This retrospective clinical study was conducted between the dates of January 2014 and January 2018 in the department of otorhinolaryngology of our hospital with 81 cases (33 females, 48 males, mean age 22.1 ± 10.1 years, interval 18–49 years) who underwent transcanal endoscopic type 1 tympanoplasty with limited elevation of tympanomeatal flap and whose Middle Ear Risk Index (MERI) scores were 1‒3. The study was approved by the Ethics Committee of the same hospital (the ethical committee nº 2018/11/05-11-E45058). The study was carried out in accordance with the Principles of the Declaration of Helsinki and Guideline for Good Clinical Practices.

Patients who underwent endoscopic type 1 tympanoplasty with limited elevation of tympanomeatal flap, were above 18 years of age, and were followed up in our clinic for at least 6 months were included in the study. Tympanoplasty types other than type 1, endoscopic tympanoplasties without elevation of tympanomeatal flap, microscopic types of tympanoplasty, patients with mastoidectomy, defects of the ossicular chain, cholesteatoma, tympanosclerosis, patients who underwent prior tympanoplasty operation and those who did not continue with follow-up on a regular basis were excluded from the study.

All patients were followed-up in our clinic for at least 12 months prior to the surgery. All patients underwent computed tomography and audiologic examination prior to the surgery. All of the patients were operated under general anesthesia according to the well-known principles of ear surgery.

Age, gender, operated ear, size of the tympanic membrane perforation, type of graft used, pathologies and status of middle ear, preoperative and postoperative audiologic test results, graft status during postoperative follow-ups, and tracking periods of all of the cases were recorded. The data were obtained by scanning the files of the patients in the hospital records system. Middle ear status of the patients were assessed beforehand using MERI that was developed by Becvarovski and Kartush.^13^ We aimed to prevent bias between groups by providing the standardization of the cases using the MERI score. Cases with MERI scores above five were excluded from the study. Tympanic membrane perforations were classified as total (100%), subtotal (>50%), below 50% (medium).

**Figure 1:**
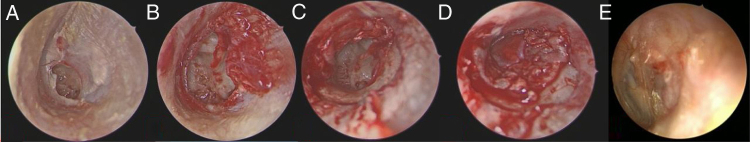
(A–E) Preoperative and postoperative surgical stages of Group A. (A) Preoperative view of tympanic membran perforation. (B) After reviving the perforation edges. (C) Limited tympanomeatal fleb elevation. (D) The grafts were placed appropriately with the over-underlay technique. (E) Tympanic membran view of postoperative 6th month).

**Figure 2:**
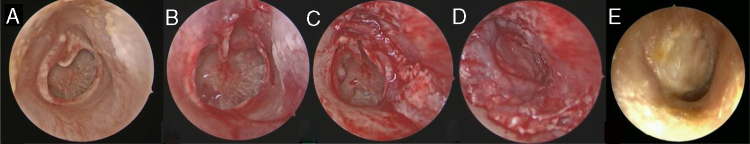
(A–E) Preoperative and postoperative surgical stages of Group B. (A) Preoperative view of tympanic membran perforation. (B) After reviving the perforation edges. (C) Limited tympanomeatal fleb elevation. (D) The grafts were placed appropriately with the over-underlay technique. (E) Tympanic membran view of postoperative 6th month).

The cases that were included in the study were divided into two groups according to the type of graft used. All cases were divided into two groups as tragal cartilage perichondrium (Group A) and temporal muscle fascia (Group B). The type of graft used in tympanoplasty was assigned according to the experience and preference of the surgeons (one surgeon used only tragal cartilage perichondrium while one surgeon used only temporal muscle fascia as grafts). All of the operations were performed by two different surgeons in the clinic in accordance with the well-established principles of ear surgery. Tympanic membrane perforation was assessed endoscopically in all cases before the extraction of grafts. In all cases the 3 mm 18 cm rigid endoscope (Karl Storz HOPKINS II®) and Karl Storz 24 INCH Full HD® monitor was used. Grafts were taken up after the edges of the perforation were debrided. In all cases, the middle ear was entered after the tympanomeatal flap was elevated 5 mm lateral to the annulus of the tympanic membrane. The middle ear ossicular chain was inspected. In Group A the grafts were obtained by taking the anterior and posterior perichondrium of the tragal cartilage, and the tragal cartilage was preserved. The obtained grafts were placed accordingly with the over-underlay technique after being shaped to fit the tympanic membrane perforation, checking the ossicular chain. The tympanomeatal flap was then repositioned (Fig. 1A–E). In Group B the grafts were obtained by supraaural incision of 2–3 cm from the temporal muscle fascia. The obtained grafts were placed accordingly to close the perforation with the over-underlay technique after being shaped to fit the tympanic membrane perforation, checking the ossicular chain. The tympanomeatal flap was repositioned (Fig. 2A–E). The supra-aural incision was sutured in accordance with the anatomical plane. The grafts were supported medially and laterally by gelfoam.

The sutures of the patients in the fascia group were removed at the postoperative first week. In all cases, the gelfoam particles that remained in the external ear canal at the postoperative third week without dissolving were aspirated to clearly inspect the tympanic membrane. The status of the tympanic membrane at the postoperative 1st, 3rd and 6th months, and the pure tone audiometry results at the postoperative 6th month were recorded. The comparison of the groups were made considering the pre- and postoperative ABG and the tympanic membrane status. The tympanic membrane being intact without retraction or lateralization, and the Air-Bone Gap (ABG) being reduced below 20 dB were evaluated as criteria of success. ABG was calculated by taking the mean value of four frequencies (0.5, 1, 2 and 4 kHz).

### Statistical analysis

For statistical analyses, NCSS (Number Cruncher Statistical System) 2007 (Kaysville, Utah, USA) software program was used. For the evaluation of the study data, descriptive statistical methods (mean, standard deviation, median, frequency, rate, minimum, maximum) were used. Student's *t*-test was used for comparison of quantitative data with normal distribution between the two groups and the Mann Whitney U test was used for the comparison of variables which did not show a normal distribution. The Paired Samples test was used to compare postoperative and preoperative evaluations. Pearson’s Chi-Square test and Fisher Freeman Halton test were used to compare qualitative data. Significance was evaluated at *p* < 0.05.

## Results

Among all of the cases, the tympanoplasty operation was performed at the right side in 44.4% (n = 36) of the patients, and at the left side in 55.6% (n = 45) of them. There were 41 cases in Group A while there were 40 cases in Group B. The follow-up period of all cases ranged between 6 and 40 months, and the mean follow-up period was 20.1 ± 8.6 months. Operation times were 54.46 ± 8.22 min in Group A and 53.34 ± 9.04 min in Group B. There were no statistically significant differences in the distributions of age, gender, groups, track lengths, MERI scores, operation times, and the sizes of perforation (all *p*-values > 0.05) (Table 1).

**Table 1:** Comparison of subject data in each group.

Variables	Group A (Perichondrium) (n = 41)	Group B (Fascia) (n = 40)	*p*
Age (years)	22.10 ± 9.26	21.68 ± 11.40	0.862^a^
Gender			0.386^c^
Females	18 (43.9%)	15 (37.5%)	
Males	23 (56.1%)	25 (62.5%)	
The side operated			0.392^c^
Right	18 (43.9%)	18 (45.0%)	
Left	23 (56.1%)	22 (55.0%)	
MERI score	2.01 ± 0.92	2.26 ± 1.08	0.390^b^
Graft intact			0.797^c^
Perfore	3 (7.4%)	4 (10.0%)	
Intact	38 (92.6%)	36 (90.0%)	
Perforation size			
Medium (25‒50)	25 (60.9%)	26 (65.0%)	0.210^a^
Total (100%)	2 (4.9%)	1 (2.5%)	0.620^a^
Subtotal (>50)	14 (34.2%)	13 (32.5%)	0.179^a^
Mean operating time (minute)	54.46 ± 8.22	53.34 ± 9.04	0.532^d^
Mean follow-up time (month)	20.1 ± 8.62	21.8 ± 13.01	0.213^d^

a Independent samples test.

b Yates continuity correction.

c Fisher’s exact test.

d Mann–Whitney U test.

The mean preoperative air-bone gap in Group A was 27.3 ± 4.7 dB, and mean postoperative air-bone gap was 14.60 ± 6.2 dB. Mean preoperative air-bone gap in Group B was 28.1 ± 6.4 dB, and mean postoperative air-bone gap was 12.5 ± 7.2 dB. There was no statistically significant difference between Group A and Group B in preoperative air-bone gap values ​​(*p* = 0.608). There was no statistically significant difference between the postoperative air-bone gap values of Group A and Group B (*p* = 0.529). In Group A, postoperative ABG values demonstrated significant decrease compared to the preoperative values (*p* = 0.0001). In Group B, postoperative air-bone gap values demonstrated significant decrease compared to the preoperative values (*p* = 0.0001). Group A and Group B did not demonstrate significant differences between postoperative improvements of air-bone gap values (*p* = 0.687) (Table 2).

**Table 2:** Comparison of ABG and the hearing gains between the two groups pre- and postoperatively.

Air bone gap	Preoperative ABG (dB)	Postoperative (dB)	*p*^a^	Gain (median)	*p*^b^
Group; mean ± SD					0.687
Group A (n = 41)	27.3 ± 4.7	14.60 ± 6.2	0.001^c^	12.90 ± 4.88	
Group B (n = 40)	28.1 ± 6.4	12.5 ± 7.2	0.001^c^	15.60 ± 6.98	
*p*^b^	0.608	0.592			

a Paired samples test. Comparison ABG between two groups pre- and postoperatively.

b Mann Whitney U test. Comparison between two groups in terms of gain.

c *p* < 0.01.

**Figure 3:**
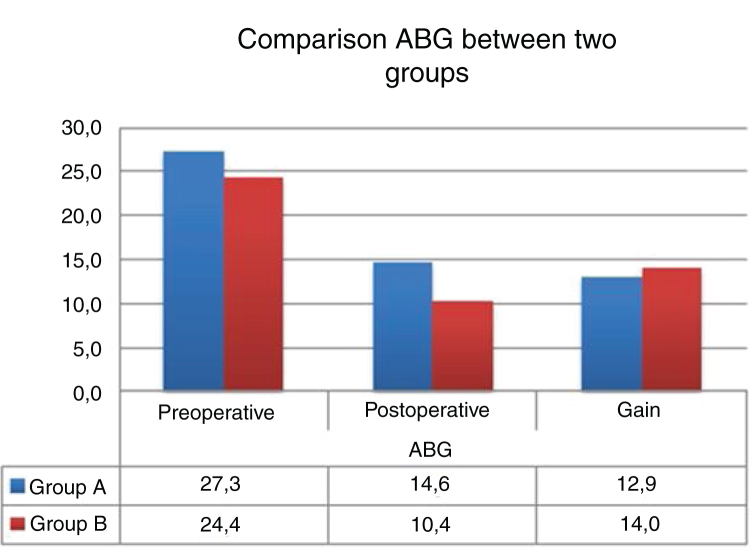
Comparison ABG between two groups.

Graft retention success was 92.6% in Group A while it was 90.0% in Group B. There was no statistically significant difference between the groups in terms of graft retention success (*p* = 0.166) (Table 3) (Fig. 3).

**Table 3:** Comparison of success rates between the groups.

	Group A (n = 41)	Group B (n = 40)	*p*
Graft retention success	92.6% (38)	90.0% (36)	0.166^a^
Hearing success (ABG ≤ 20 dB)	87.80% (36)	92.5% (37)	0.422^a^

a Fisher’s exact test.

## Discussion

Developments related to tympanoplasty, one of the most common otologic surgical procedures in otology clinics, still continue.^1^, ^3^ Otologists are exploring new techniques and methods to increase tympanoplasty success and to reduce the incidence of revision surgery. With the advancement of the technological developments in the medical field, it has become possible to shorten the duration of the operations, to improve the success of the operations, and to improve the quality of life after the operations. With the initialization of the usage of endoscopic technique in middle ear surgery, the concept of minimally invasive surgery was developed. Studies conducted have identified endoscopic ear surgery as reliable with minimal complications and morbidity.^2^, ^3^

The main factor affecting the graft retention success in tympanoplasty is the blood supply system and neovascularization that provides the graft nourishment.^10^ In situations where the arterial blood supply of the graft becomes disrupted, failure will be inevitable. In a study in which Applebaum and Deutsch^14^ investigated the dynamic vascularization of tympanic membrane by fluorescein angiography, it was reported that the arterial blood supply of the posterior quadrant of the tympanic membrane originated mainly from the mallear artery, and that the posterior tympanic membrane quadrant had a richer perfusion than the anterior quadrant. The same authors detected that the blood flow to the anterior tympanic membrane quadrant came mainly from blood vessels branching from the annulus, and that it was consistently less than that of the posterior quadrant. In their study, Hellström et al.^15^ reported that after being incubated in the external carotid artery, in the early stages, the dye first appeared in the mallear and annular vessels. In the studies conducted it was presumed that in cases with limited elevation of the tympanomeatal flap, the blood perfusion would be less disrupted and the success of graft retention would be higher. With the continuation of blood perfusion, wound healing would be rapid and graft retention success would increase. Limited incisions would cause less disruption in the continuity of the annulus and the tympanomeatal flap. Furthermore, it was suggested that this technique might prevent complications related to the dissection of the annulus and might reduce residual perforations, especially at the anterior aspect.^10^, ^11^ In the endoscopic cartilage tympanoplasty performed by Kaya et al.,^10^ it was reported that the limited elevation of tympanomeatal flap was a secure method with minimal complications.

Ayache,^11^ according to the size and location of the perforation in endoscopic tympanoplasty, defined different varieties of incisions. The cited author reported that a radial incision of up to 80% of the annulus would be sufficient for large and more anterior perforations. Although limited tympanomeatal flap elevation is an important factor affecting graft success, its use for the evaluation of limited middle ear structures and ossicular chain system may lead to reduced application of it by the surgeons. Furthermore, because endoscopic tympanoplasty has a certain learning curve, it may lead to a longer duration of surgery at the beginning.^5^, ^16^ However, despite the involvement of a limited incision, owing to both the flexibility of the graft and the flexible nature of the tympanomeatal flap, these restrictive factors can be relatively less challenging. With the experience of the surgeon, these limiting factors will be eliminated over time.

In this study, the success of grafts were compared in endoscopic tympanoplasty performed with limited elevation of tympanomeatal flap. It was observed that perichondrial and fascial grafts had similar anatomic and functional success in endoscopic tympanoplasty performed with limited elevation of tympanomeatal flap. Graft retention success was consistent with the results of previously performed endoscopic and microscopic tympanoplasty operations.^4^, ^6^, ^17^, ^18^ Tragal cartilage perichondrium and temporal muscle fascia could be safely preferred as grafts in endoscopic tympanoplasty. In addition, for all perforation types, it was observed that the limited elevation of a tympanomeatal flap was not a restrictive factor for surgery. In surgical operations performed in accordance with the basic principles of endoscopic tympanoplasty, preferring the limited elevation of tympanomeatal flap did not create any negative results in terms of surgical success. Furthermore, in our previous study on transcanal endoscopic tympanoplasty in pediatric patients, tympanometatal flap elevation was not performed.^19^ The graft retention success rates were 94.7% and 90.5% with perichondrium and fascia grafts in the previous study. Success rates in the present study were relatively lower than in previous studies. This condition may explain the importance of limited elevation of tympanomeatal flap. We think that the graft retention success rates are decreased with the amount of limited elevation of tympanomeatal flap during the endoscopic tympanoplasty.

The cases reporting their results throughout a relatively long term (postoperative 6 months) was among the strengths of this study. In the literature there was no prior study comparing the success of two grafts placed with limited tympanomeatal flap elevation in endoscopic tympanoplasty and the fact that this was the first study on this subject renders the study interesting for the reader. Other strengths included strict implementation of the inclusion criteria, and the fact that there was no data which would lead to bias among the study groups in demographic characteristics to negatively affect the study. Additionally, providing the standardization of the cases using the MERI score enabled the prevention of bias among the groups.

Although this study reported very valuable data, there were some limitations. The retrospective nature of the study and the relatively small number of cases were the main limitations. Also the inclusion of two different surgeons in the study was considered as a limitation. However, the fact that both surgeons had more than 5 years of experience in ear surgery and operated in accordance with the endoscopic otologic surgical principles relatively reduced the negative impact of having different surgeons on surgical outcomes. In future, further prospective randomized studies with higher number of patients will be required.

## Conclusion

In accordance with the results of this study, we believe that both tragal cartilage perichondrium and temporal muscle fascia, and also limited tympanomeatal flap elevation in endoscopic tympanoplasty are all eligible for safe and successful surgery.

## Conflicts of interest

The authors declare no conflicts of interest.
